# Survey of knowledge, practice, and associated factors toward home management of childhood fever among parents visiting Gondar health facilities in 2022

**DOI:** 10.3389/fped.2024.1100828

**Published:** 2024-03-01

**Authors:** Nega Tezera Assimamaw, Almaz Tefera Gonete, Bewuketu Terefe

**Affiliations:** ^1^ Department of Pediatrics and Child Health Nursing, School of Nursing, College of Medicine and Health Sciences, University of Gondar, Gondar, Ethiopia; ^2^ Department of Community Health Nursing, School of Nursing, College of Medicine and Health Sciences, University of Gondar, Gondar, Ethiopia

**Keywords:** fever, under-five, parents, knowledge, practice, Ethiopia

## Abstract

**Background:**

Fever is a typical symptom of many sicknesses, but for children under the age of five, fever can have devastating consequences and represents a source of worry for parents. To the best of our knowledge, no research on home management of fever in children has been conducted in Ethiopia. We aimed to assess knowledge, practices, and associated factors towards home management of childhood fever among parents visiting Gondar Town health facilities in 2022.

**Method:**

This multicenter institutional-based cross-sectional study was conducted in Gondar public health facilities from June 1st—June 30th, 2022. Participants were fathers and mothers of children aged 0–5 years. A stratified random sampling technique was used. Data were collected through face–to–face interviews using a pretested structured questionnaire.

**Results:**

Approximately, 40.2% (95% CI: 35.5%, 45.2%) of parents had good knowledge and only 12.8% (95% CI: 9.7–15.8) of parents practiced home fever management. Being married [Adjusted odds ratio [(AOR) = 2.1 (1.2, 3.2)], having a primary or higher level of education [AOR = 2.4 (1.17, 4.9)] [AOR = 2.0 (1.02–4.6)], respectively, and number of children [AOR = 1.8 (1.63, 2.03)] were factors associated with parental knowledge. Likewise, being married [AOR = 3.05 (2.27.50–3.83)], receiving counseling from health care providers [AOR = 2.12 (1.53–3.32)], and being male [AOR = 2.03 (1.50–3.00)] were significant predictors of practice.

**Conclusion:**

Inadequate levels of knowledge and numerous irrational practices related to home fever management were predominant among parents, which needs to be addressed. Evidence-based health education is essential for parents to enhance their level of knowledge and practice to effectively treat fever at home.

## 1 Background

Fever is a common symptom seen in children with various illnesses (1–5). Fevers lasting more than three days warrant visits to the emergency room, which can lead to overcrowding and higher healthcare costs (6), and about 70% of children under the age of 18 consult a doctor for fever-related illnesses (7). Fever is not a disease itself, but rather a symptom or sign of an underlying condition (8). It can be considered a threat-adaptive physiological response (9–13).

There is no doubt that parents may experience anxiety when it comes to the potential side effects of fever. Although fevers are generally regarded as a healthy response, there can be concerns about complications such as convulsions, dehydration, brain injury, and even death in rare cases (14, 15). In addition, most parents are unaware of the definition of fever, its symptoms, or how to treat it (16, 17).

It is possible to define fever or pyrexia based on its pathophysiology and clinical purpose. Pathophysiologically, exogenous pyrogens cause fever by causing host cells (mainly macrophages) to produce and release endogenous pyrogens, which are involved in some immune system functions. Endogenous pyrogens, which are substances produced by the body in response to infection or inflammation, can reach the hypothalamic thermoregulatory center. Specifically, they can target the Organum Vasculosum of the Lamina Terminalis (OVLT) in the hypothalamus. This triggers the production of prostaglandins, with prostaglandin E2 (PGE2) being the most important one. PGE2 acts on the hypothalamus to raise the body's temperature set point (10, 18–20). To start the febrile response, these raise the thermostatic set point (21). According to the World Health Organization (WHO), the Society of Critical Care Medicine, and the Infectious Disease Society of America (IDSA), among others, equivalent rectal temperatures of 38°C (100.4°F) or axillary temperatures of 37.5°C (99.5°F) in both adults and children indicate fever (22–25). Hence, a 10%–12.5% increase in metabolic rate is required for a 1°C increase in body temperature (26).

Treatment for febrile seizures (FS) is generally not advised because most occurrences are benign and self-limiting (27);that is, 80% are mild febrile convulsions with no lasting effects (28). However, parents believe that FS can cause brain damage and death, and this belief has been identified as the primary driver of parental dread of fever (15, 29, 30). In addition, numerous studies reported that 14.4%–21% of parents in Western nations such as the USA and Australia believe that fever damages the brain, and fever phobia is still common in Asian nations like Taiwan and Singapore, where 68.8%–77.7% of parents hold this belief (31–34).

In certain Sub-Saharan African nations, fever constituted more than 50% of visits to pediatric outpatient clinics (35).

According to Rajan Arora and Prashant Mahajan, fever in children is most commonly caused by infections, although it can also be caused by immune-mediated, inflammatory, or neoplastic disorders. “Fever without a source” occurs when the reason for the fever cannot be determined through history and physical examination (36).

Different cultures have different levels of knowledge regarding fever (37), appropriate antipyretic use (38, 39), level of fever phobia (40), and, in general, parental fever care (34, 37, 41). However, fever anxiety can still be found in people from all cultures, including those from Europe. In European cultures, where access to healthcare is generally widespread, individuals may still experience anxiety related to fever (40). Moreover, approximately half of all parents use antipyretics when the temperature is below 38 degrees Celsius, or use inappropriate antipyretic doses or supra-therapeutic amounts of paracetamol and ibuprofen. Four decades ago, Barton Schmitt coined the term “fever phobia” in his classic study to characterize parents' irrational anxieties about fever (29). The 2021 version of the NICE (National Institute for Health and Care Excellence) guideline regarding fever in children does not recommend tepid sponging, which involves using water to reduce body temperature. It is recommended not to over-wrap children with fever, and paracetamol or ibuprofen should be considered if the child seems distressed. Furthermore, if one medication fails to alleviate the child's distress, a switch to another agent may be considered (for example, from paracetamol to ibuprofen or vice versa), and paracetamol and ibuprofen should not be administered simultaneously (42).

Certain studies have highlighted that educational level, financial class, and cultural background were the most important factors in childhood fever knowledge and management (43–45).

In 2019, M. Kelly and his colleagues ran a randomized control trial (RCT) to determine the effectiveness of information leaflets in raising parental awareness on childhood fever. According to the findings, enhancing parental knowledge of fever and suitable management options (46). There is no recent evidence on parents' knowledge and practice of home management of fever and its contributing factors in Ethiopia. Therefore, this study aimed to assess the knowledge, practices, and associated factors toward home management of childhood fever among parents visiting Gondar town health facilities, in Amhara regional state, northwest Ethiopia, in 2022. Thus, this study can be used as a baseline by health policymakers to help design strategies.

## 2 Methods

### 2.1 Study design and period

An institutional-based cross-sectional quantitative study was conducted from June 1st, 2022—June 30th, 2022.

### 2.2 Study area

The research was conducted in Gondar's public health facilities. According to the Federal Democratic Republic of Ethiopia's Central Statistical Agency's (CSA) official population projection of Ethiopian cities and towns in 2015, the town has an estimated population of 323,900 people (47). Currently, one comprehensive specialty hospital and eight health centers provide health care services to the community in the municipality, totaling nine public health facilities. According to the Gondar municipal health office, 652,940 patients visit all nine governmental health institutions in the town each year, with 21,527 of them being children under the age of five (48).

### 2.3 Sources population

All parents of children who visited the governmental health facilities in Gondar town were a population source.

### 2.4 Study population

Parents of children and those available during data collection at governmental health facilities in Gondar town were the study population.

### 2.5 Inclusion criteria

All parents who have children and visit the governmental health facilities in Gondar town were considered for inclusion.

### 2.6 Exclusion criteria

Parents of children found in Gondar metropolitan city's health institutions with serious illness conditions or with hearing loss problems (with no interpreter of sign language) were excluded from the study.

### 2.7 Sample size determination

The sample size was calculated using a single population proportion formula based on the following statistical assumptions:

Because there is no previous research conducted on this topic in Ethiopia, we considered *P* = 50%, 95% confidence level, and a 5% margin of error.


$$\;n=\frac{Z\;(\alpha \;/2)2\;\;p\;(1-p)}{(d)2}$$

Where:

*n* = the desirable sample size

Z (*α*/_2_) = the critical value at the 95% level of significance (1.96)

P = proportion of knowledge or practice.

d = margin of error


$$n=\frac{(1.96)2\times 0.5\times 0.5}{(0.05)2}=384$$

After adding a 10% non-response rate, the total sample size was 423.

### 2.8 Sampling technique and sampling procedure

**Figure 1:**
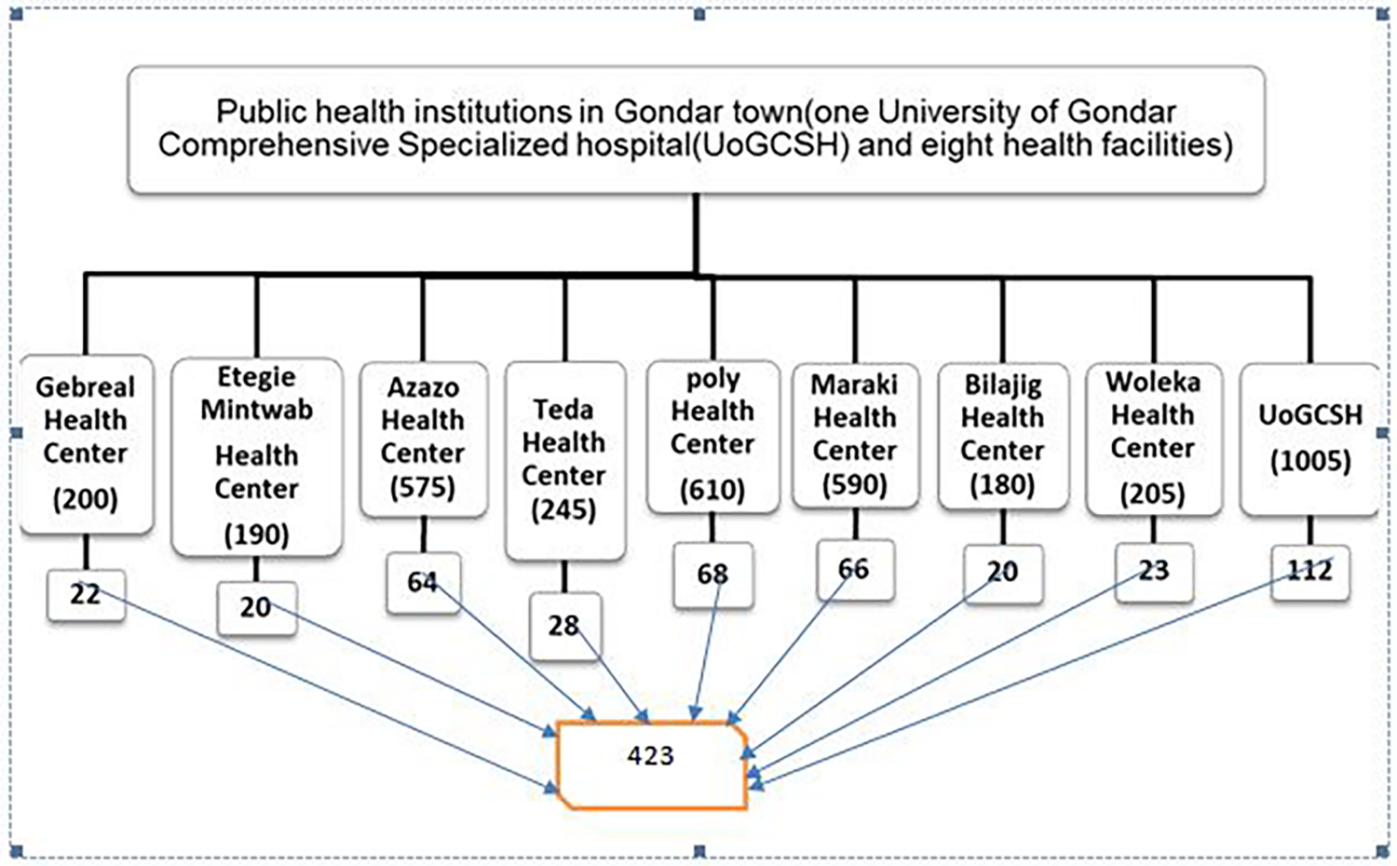
Schematic diagram of the sampling procedure for the study population in Gondar town governmental health facilities, Amhara regional state, Ethiopia 2022.

To obtain a representative sample from the town's eight public health institutions and one comprehensive specialized hospital, a stratified random sampling technique was used. The study covered all healthcare facilities, and the total sample size was proportional. During the study period (one month), a 3,800 customer flow rate was predicted. To estimate the sampling interval (k), the determined sample size (n) of respondents (i.e., 3,800/423 = 8.9) was divided by the anticipated number of parents visiting the public health facilities during the study period (N). As a result, one in every nine parents was chosen using a systematic random sampling procedure until the required sample was reached (Figure 1).

### 2.9 Data collection tools and procedures

The data were collected using structured, pretested, and interpreted face-to-face interviewer-administered questionnaires. All questions were derived from various sources (15, 49, 50). This questionnaire broadly encompassed three sections. Section 1 comprised the characteristics of the study participants (age of the child in months, sex of the child, age of parents/guardians in years, sex of the parent, marital status, ethnicity, level of education, occupation, religion, monthly income status, relationship to the child, and number of children). Section two included 16 questions to evaluate the knowledge of parents regarding the home management of fever. Section three consisted of nine questions for an assessment of the practice of parents in the home management of childhood fever.

### 2.10 Variables of the study

#### 2.10.1 Dependent variables

• Knowledge of home management of fever (good/poor)
• Practices on home management of fever (good/poor)

#### 2.10.2 Independent variables

**Sociodemographic-related factors:** age of the child in months, sex of the child, age of parents/guardians in years, sex of the parent, marital status, ethnicity, level of education, occupation, religion, monthly income status, relationship to the child**,** and number of children.

### 2.11 Operational definitions

The term “fever” is defined as “an elevation of body temperature above the normal daily variation” (51). Fever among children under 5 years of age is often defined as a temperature of 100.4°F (38°C or higher) (52). When the body's temperature exceeds 40°C, it is referred to as a high fever (53), and high fever is best treated by giving antipyretics like paracetamol or ibuprofen (54, 55).A documented oral, ear, and forehead temperature of 37.8°C or higher (also known as the “rectal equivalent”) was considered to be fever. By adding 0.2°C to the oral temperature and deducting 0.6°C from the axillary temperature, a rectal equivalent temperature was determined (56).

Parents were classified as having good knowledge if their responses to knowledge-related questions were greater than or equal to the mean, whereas parents with answers that fell below the mean were classified as having poor knowledge.

Parents who scored above or equal to the mean value on practice-related questions were considered to have good practices, whereas those who scored below the mean value on knowledge-related questions were considered to have poor practices.

### 2.12 Data quality control

The instrument was translated into the Amharic language (local language), and to check the tool's consistency, it was translated back to English to ensure data quality. For consistency of comprehension of the survey tool and modification, the structured questionnaire was pre-tested on 5% of the total sample outside the chosen health facility. Before beginning data collection, the recruited data collectors received one-day training on the purpose, confidentiality of information, relevance of the study, respondent's rights, pretest, informed consent, and interviewing techniques.

### 2.13 Data processing and analysis

Data were coded, cleaned, and entered into Epi-Info version 7, and then exported to SPSS version 20 for statistical analysis. Sociodemographic and other study participant variables were summarized using descriptive statistics (frequencies and percentages). To show the degree of connections between the explanatory and outcome variables, binary logistic regression analysis was used. In the multivariate analysis, only factors with *P* < 0.25 in the bivariate analysis were considered. With a 95% confidence interval, *P* < 0.05 on the multivariate analysis was considered statistically significant.

### 2.14 Ethical considerations

Ethical clearance was issued from the school of nursing research ethical review committee on behalf of the University of Gondar. Each health facility's head or coordinator received a formal letter outlining ethics and collaboration, and the head of the health facility approved and forwarded a permission letter to the unit team leader/coordinator. The unit team leader was permitted to collect data. Participants in the study provided their informed consent after being informed of their opportunity to opt out of the study. Data were coded to maintain the confidentiality of the information for each study participant.

## 3 Result

### 3.1 Sociodemographic characteristics of the respondents

There were 423 participants, of which 95% (402) were female and gave a 100% response rate. The study participants' ages ranged from 26 to 35 years old, with a mean (SD) age of 30.28 ± 6.534 years. Of the children, 42.6% (180) were under the age of six months, which is the age group where the majority of them fell. More than half of the children, or 239 of them, were female, making up 56.5% of the total. Less than one-third, 33.5% (106), of the study participants had diplomas or a formal education, and the maximum percentage, 75.5% (319), of the study participants were Amharan in ethnicity. Approximately 85.8% (363) of the study participants were married.

Regarding occupational status, about 41.4% of the study participants were housewives, and 86.1% (364) were orthodox religious followers. Most study participants, 79.4% (336), earned a monthly income of 500–2,500 Ethiopian Birr. In addition, 95.7% (405) of children had a relationship with their biological father /mother and more than one-third, 32.9% (139), of the study participants had ≥3 children (Table 1).

**Table 1:** Sociodemographic characteristics of the participants in Gondar town health facilities, 2022 (*n* = 423).

Variables	Frequency	Percentage (%)
Age of the child in months		
0–5	180	42.6
6–23	154	36.4
24–35	89	21
Sex of the child		
Male	184	43.5
Female	239	56.5
Age of parents/guardians in years		
18–25	100	23.6
26–35	196	46.3
36–45	71	16.8
>45	56	10.9
Sex of the parent		
Female	402	95.0
Male	21	5.0
Marital status		
Single	14	3.3
Married	363	85.8
Divorced/Separated	36	8.5
Widowed	10	2.4
Ethnicity		
Amhara	319	75.5
Tigre	56	13.2
“Kimant”	48	11.3
Level of education of mothers		
No formal education	106	25.1
Primary education	98	23.2
Secondary education	77	18.2
Diploma and above	142	33.5
Level of education of fathers		
No formal education	71	16.8
Primary education	89	21.0
Secondary education	126	29.8
Diploma and above	137	32.4
Occupation		
Housewife	175	41.4
Student	21	5.0
Farmer	5	1.2
Merchant	45	10.6
Daily laborer	19	4.5
Government employee	99	23.4
Private worker	59	13.9
Religion		
Orthodox	364	86.1
Muslim	54	12.8
Protestant	5	1.2
Monthly income		
No monthly income at all	87	20.6
500–2,500 ETB	156	36.9
2,500–5,000 ETB	124	29.3
>5,000 ETB	56	13.2
Relationship to the child		
Biological parent	405	95.7
Grandparent/caregiver	18	4.3
Number of children		
1	131	31.0
2	153	36.2
≥3	139	32.9

### 3.2 Knowledge of parents about home-based management of fever

Parents as a whole had a knowledge level of 40.20% (95 percent confidence interval) (35.5–45.2). In a similar vein, 33.8% (143) of survey participants recognized what fever was. 18.4% (78) of the total responders who were aware of the definition of fever could recognize it. Knowledge of the appropriate temperature measurement: Of the study's participants, 27% (114) were aware of the proper normal temperature readings, while 22.7% (96) were aware of the typical range of body temperature measurements. 71.4% (302) of the study's total participants expressed concern when their child's body temperature increased.

Similarly, of the total surveyed participants, 238 (56.3%) were aware of the causes of fever, including infection (31.7%), stress (22.0%), immunization (30.5%), and seasonal allergies (13.5 percent). A total of 236 study participants—or 55.8%—knew how to measure their body temperatures. 55.1 percent of study participants were familiar with measuring their body temperatures under their arms or in their axillae at home (236).

Regarding the technique for taking temperature readings and how frequently they were taken, 64.1% (271) and 25.1% (106) of the study's participants used their hands and the intervals of 15–30 min for temperature readings and body temperatures, respectively. However, only 26.2% of the survey participants reported managing their fever at home (111). Nevertheless, most of the research participants—12.5% (53)—provided antibiotics to treat fevers, whereas 6.4% (27) gave acetaminophen. In addition, treatments include cold sponging (64.5%), using an ice pack (15.4%), and just using medications (12.3 percent). Most study participants, 72.8% (308) and 12.3% (52), utilized the precise measurement spoon or syringe that comes with the medication to administer drugs, respectively (Table 2).

**Table 2:** Knowledge of parents on home-based managements of fever in Gondar town health facilities, 2022 (*n* = 423).

Variables	Frequency	Percentage (%)
Know about fever		
Yes	151	35.7
No	272	64.3
Fever means		
Body temperature >38 degrees Celsius	143	33.8
Body temperature >37.5 degrees Celsius	168	39.7
Body temperature <37.5 degrees Celsius	57	13.5
Specify if any	55	13.0
Participants who know about normal temperature measurement reading		
Yes	114	27.0
No	309	73.0
Participants who know the range of normal body temperature		
32°C–34°C	6	1.4
34°C–35°C	3	0.7
35°C–36.2°C	9	2.1
36.2°C–37.5°C	96	22.7
The feeling of parents when their child's body temperature raises		
Worried	302	71.4
No response	62	14.7
Take to health institution	45	10.6
Consult health professionals	13	3.1
Other	1	0.2
Parents who know about the cause of fever		
Yes	238	56.3
No	185	43.7
The specific cause of fever		
Infection	134	31.7
Stress	93	22.0
Vaccination	129	30.5
Seasonal	57	13.5
Others	10	2.4
Knowing how to measure body temperature at home		
Yes	236	55.8
No	187	44.2
Site of temperature measurement		
Armpit/axillary	233	55.1
Oral/Mouth	67	15.8
Anus/rectal	64	15.1
Others(skin)	59	13.9
Method of temperature measurement		
Hand	271	64.1
Electronic thermometer	5	1.2
Mercury-in-glass thermometer	7	1.7
I do not check my child's temperature	14	3.3
I do not know	125	29.6
Frequency of measuring temperature		
Less than 15 min	98	23.2
15–30 min	106	25.1
30 min–1 h	82	19.4
1–2 h	16	3.8
More than 2 h	9	2.1
Knowing how to manage fever at home		
Yes	111	26.2
No	312	73.8
Medications are given to the child at home		
Acetaminophen	27	6.4
Ibuprofen	9	2.1
Aspirin	7	1.7
Antibiotics	53	12.5
Other	15	3.5
Remedies used in addition to drugs		
Cold sponging	273	64.5
Ice pack	65	15.4
Tepid sponging	7	1.7
I use drugs only	52	12.3
Any other remedy	26	6.1
Site of medication administration		
Oral	308	72.8
Rectal	113	26.7
Tools used to administer the medication		
Teaspoon	32	7.6
Specific measurement spoon or syringe provided with the drug	52	12.3
Measuring spoon or syringe of other drugs	27	6.4
Received advice about fever		
Yes	178	42
No	245	58

**Figure 2:**
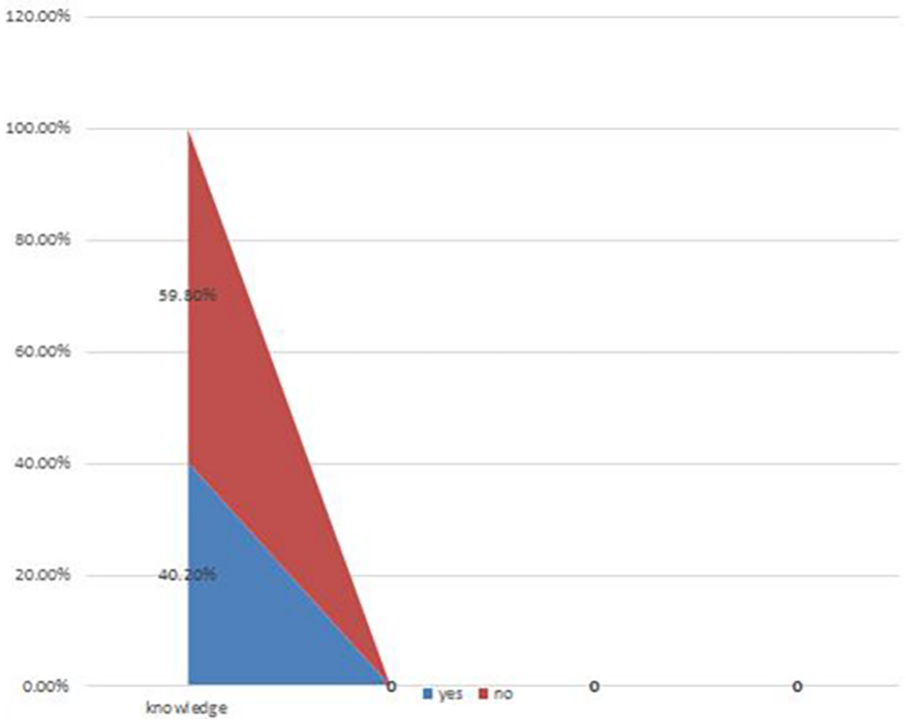
Knowledge of parents on home management of fever in Gondar city health institutions, Ethiopia (*n* = 423).

Knowledge of parents on home management of fever in Gondar City Health Institutions, Ethiopia (Figure 2).

### 3.3 Practice of parents' home-based management of fever

Overall, 12.8% of parents practiced managing fevers at home (95 percent confidence interval (9.7–15.8). Only 2.6% (11) of the survey participants said that they regularly measured their body temperatures at home, and 2.8% (12) reported having thermometers, both glass (1.7%) and electronic (batter, thermometer) ones (1.2 percent). Only 23.2% (98) of the participants used a proper temperature measurement technique other than a thermometer, such as using the dorsum of the hands (23.2 percent).

When a child has a fever, parents treat them immediately with wet sponging (22.5%), cold water baths (58.2%), analgesics (9.2%), antipyretic medications (3.8%), homeopathic remedies (59%), and doctor consultations (0.2 percent). More than one third, 34.3% (145), of the survey participants also phoned doctors when their body temperatures climbed to 43°C. The majority of participants, 63.6% (269), did not know when therapy should be delivered or the range of body temperatures.

Appropriate antipyretic medication was administered based on prior pediatrician advice (42.1%) and pharmacist consultation (31.4%). Additionally, 43% (182) of the trial participants calculated fever-lowering medicines based on prior pediatrician advice (Table 3).

**Table 3:** Practice of parents on home management of fever in Gondar town health facilities, 2022 (*n* = 423).

Variables	Frequency	Percentage
The practice of measuring body temperature at home		
Yes	11	2.6
No	412	97.4
Possession of thermometer		
Yes	12	2.8
No	411	97.2
Apparatus practically used to measure body temperature		
Glass thermometer	7	1.7
Electronic (battery, thermometer)	5	1.2
Have no idea	411	98.1
Mechanisms used to measure body temperature apart from the thermometer		
Using the dorsum of the hand	98	23.2
Using palms	152	35.9
Using fingers	69	16.3
Using ventral hand	104	24.6
Immediate response to child's fever		
Wet sponging	95	22.5
Cold water bath	246	58.2
Gives antipyretic	39	9.2
Gives antipyretic medicine	16	3.8
Gives homeopathy medicine	25	5.9
Consult doctor	1	0.2
When treatment is administered, if body temperature is		
36_C	15	3.5
37_C	19	4.5
38_C	90	21.3
39_C	24	5.7
40_C	6	1.4
41_C	269	63.6
When a doctor is called?		
38_C	93	22.0
39_C	59	13.9
40_C	41	9.7
41_C	0.1	0.2
42_C	1	0.2
43_C	145	34.3
The right antipyretic drug based on		
Previous advice from the pediatrician	178	42.1
Consultation of pharmacist	133	31.4
Consultation of other persons	39	9.2
Information collected by media	13	3.1
I decide by myself	40	9.5
I call my pediatrician	20	4.7
Calculation of fever-lowering agents		
Previous advice from the pediatrician	182	43.0
Reading the package leaflet	146	34.5
Consultation with the pharmacist	32	7.6
Media	8	1.9
I decide by myself	35	8.3
I call my pediatrician	20	4.7

Factors associated with parents' knowledge of home management of fever (Table 4).

**Table 4:** Bivariable and multivariable analyses of factors associated with parents' knowledge on home management of child hood fever in Gondar town health facilities, 2022 (*n *=* *423).

Variables	Knowledge		COR (95%)	AOR (95%)
	Good	Poor		
Sex of the child				
Male	81 (19.1%)	103 (24.3%)	0.29 (0.6–1.14)	1.41 (0.97–1.83)
Female	89 (21%)	150 (35.5%)	1	1
Marital status				
Married	102 (24.1%)	261 (61.7%)	0.39 (0.3–3.7)	2.08 (1.2–3.2)
Single	30 (7.1%)	30 (7.1%)	1	1
Level of education				
No formal education	34 (8%)	72 (17%)	1	1
Primary education	47 (11.1%)	51 (12.1%)	0.92 (0.6–1.7)	2.4 (1.17–4.90)
Secondary education	26 (6.2%)	51 (12.1)	1.7 (0.9–3.2)	1.3 (0.71–1.6)
Higher education	102 (24.1%)	40 (9.5%)	1.13 (0.6–2.2)	2.0 (1.02–4.6)
Number of children				
1	75 (17.7%)	56 (13.2%)	1	1
2	80 (19%)	73 (17.25%)	1.22 (1.12–2.6)	0.75 (0.33–1.6)
≥3	60 (14.2%)	79 (18.7%)	1.75 (1.60–1.96)	1.79 (1.63–2.03)

Bold values denote the significant variables.

Factors associated with the practice of parents in the home management of fever (Table 5).

**Table 5:** Bivariable and multivariable analyses of factors associated with parents' practice on home management of child hood fever in Gondar town health facilities, 2022 (*n *=* *423).

Variables	Practice		COR (95%)	AOR (95%)
	Good	Poor		
Sex of the child				
Male	104 (24.6%)	80 (18.9%)	2.18 (1.55–3.34)	2.03 (1.50–3.00)
Female	90 (21.28%)	149 (35.22%)	1	1
Marital status				
Married	334 (78.96%)	29 (6.9%)	13.4 (0.8–13.5)	3.05 (2.27–3.87)
Single	28 (6.6%)	32 (7.6%)	1	1
Level of education				
No formal education	7 (1.7%)	99 (23.4%)	1	1
Primary education	19 (4.5%)	79 (18.7%)	1.3 (0.4–4.0)	1.4 (0.4–4.5)
Secondary education	11 (2.6%)	66 (15.6%)	0.4 (0.14–1.0)	1.03 (0.12–1.97)
Higher education	11 (2.6%)	59 (14%)	0.5 (0.2–1.6)	0.7 (0.2–2.1)
Have you received advice about fever from the health provider				
No	182 (43%)	90 (21.3%)	1	1
Yes	73 (17.3%)	78 (18.4%)	2.16 (1.57–3.38)	2.12 (1.53–3.32)

Bold values denote the significant variables.

## 4 Discussion

This study examined knowledge, practices, and associated factors of fever home management among parents of children under five years of age. A pervasive range of childhood diseases are accompanied by fever; most of them are managed at home before visiting a nearby health care facility. If the fever is left untreated, it can result in numerous complications such as brain damage, dehydration, seizure, and organ damage (liver and kidney), which both the life of the child and the family and community at large.

Our findings regarding the knowledge of parents about childhood fever home management revealed that less than half of the participants had good knowledge (40.20%; 95% CI, 35.5%–45.2%). This insufficient level of knowledge depicts a very high public issue that demands the stakeholders' due attention. Our finding is consistent with different studies conducted in Germany (30.6%), Saudi Arabia (38.4%), and Ghana (43%) (57–59). However, our findings show a lower level of parents' knowledge than studies conducted in different countries in India (95%), the Netherlands (88%), Korea (70.64%), Ireland (63%), and France (61%) (2, 52, 60–62). The difference in developed countries is that two thirds of the participants had higher educational levels (58, 60), but in this study, only one-third of participants had higher educational status (**see** Table 1), and highly educated participants are expected to have the ability to choose quality health services and seek healthcare service early for their febrile children (46). In addition, highly educated participants are expected to have enough income to afford healthcare services and different basic goods, which are needed for best survival (63). Our findings showed that parents' level of knowledge is relatively higher than studies conducted in Taiwan and Turkey, which were 19% and 18% (34, 64), respectively., The difference could be the small sample size in Turkey and the higher level of anxiety and dissatisfaction about the information given by health care providers in Taiwan.

Our results showed that only 12.8% (95% CI: 9.7%–15.8%) of parents practiced managing fevers at home, which was lower than studies conducted in different countries (Palestine 78%, Korea 75.93%, and India at 27%) (3, 60, 61). A possible reason could be the low level of socioeconomical status of our study participants compared with those countries. A low level of socioeconomical status hinders the parents from accessing quality health care services and even from homemade fever managements because they cannot afford to buy a thermometer and might not have knowledge on some home remedies such as calf wrap. This involves applying cool or cold compresses to the calf muscles. The cool or cold temperature of the compresses can cause vasoconstriction (narrowing of blood vessels) in the peripheral areas, such as the calves. This vasoconstriction can help to divert blood away from the skin's surface and reduce body heat, thus it can prevent side effects including shivering, crying, agitation, and discomfort (65) In addition, the total domestic growth per capita is much greater in India (3,173,397.59 million US$ GDP of India and 1,798,583.92 million US$ GDP of Korea) compared to Ethiopia's GDP of 111,271.11 million US$ (66). Furthermore, the survival of children in underdeveloped countries depends on the family's and community's ability to access the basic needs of life (67).

In the current study, marital status, level of education, and number of children were associated with parents' knowledge. Parents' levels of knowledge regarding fever home management was 2.1 times higher among married parents than their unmarried counterparts. Married parents are estimated to have possible support from spouses both in terms of income generation and physical support for their febrile child (68). Married parents are also more responsive to caring for their children because they are mature enough in decision-making and resources. This is not consistent with other previous studies conducted in different countries that reported that none of the parent or child variables were found to predict accurate antipyretic usage or parental antipyretic knowledge (52, 62, 69, 70).

Parents who have more than or equal to three children increase the likelihood of fever home management knowledge 1.8 times higher than parents whose number of children is less than three. This study has similar finding to a study conducted in Jordan (71, 72). A possible reason for increased knowledge in parents with more children may be their previous knowledge and experience of effective fever management. For children with older siblings, it is likely that parents use their earlier experiences with the elder child for effective management of the present child and make better-informed decisions (61, 73); these scenarios indicated a situation where the parents would be expected to have more life experience.

Moreover, the level of knowledge doubled among parents who were more educated than their counterparts. This result corresponds with studies conducted in Canada (2, 73). Enarsonet al. illustrated that parents with a high level of educational status had a lower level of apprehension and revealed less fever phobia (73) because parents who were more worried about their fever were not able to effectively treat their febrile child (34). A higher level of education plays a significant role in elucidating child health outcomes; educated personnel are estimated to have positive health-seeking behavior, acquire crucial health-related information, and enact home-based and different management modalities provided by health care providers correctly. Likewise, education is a source of income/wage that is crucial to leading a healthy life (74, 75).

Received counseling from health care provider, sex of child, and marital status were determinants of parental fever home management practice. Parents who received counseling from health care providers had better practice skills in fever home management than parents who did not receive medical advice. This agrees with a study conducted in Morocco (75), where parents who had received advice from health care professionals were more knowledgeable on how to manage their febrile children (2), how to prevent further complications, and how to seek healthcare services early when the child did not respond to home-based treatments in addition to knowing adverse medication effects (60).

The likelihood of good practice among parents whose children were male was 2,03 times more than their counterparts, which is supported by the result reported in Malaysia (76). Moreover, in our report, married parents were more knowledgeable, which is an essential predictor of successful fever home management (61). Even though married parents are expected to share their responsibility for each other, which is crucial to lead a healthy life, there are no reports supporting this suggestion (59, 62, 71).

## 5 Strengths and limitations

The study was conducted in one city in the country, which limits the generalizability of the outcomes to all parents in the country. Future research on the topic must include a representative sample of parents from the whole country to overcome this limitation, which is similar to the limitation of a cross-sectional study. Despite these limitations, the study can be used with caution as a baseline for the development of interventions to raise the awareness of parents on the identification and management of childhood fever.

## 6 Conclusion

Marital status, number of children, and a higher level of parental education were significantly associated with parental knowledge. Likewise, receiving counseling from a health care provider and the sex of the child was a substantial predictor of good fever home management practice. Inadequate levels of knowledge and numerous irrational practices related to fever home management were predominant among parents, which needs due attention. Evidence-based health education is essential for parents to enhance their level of knowledge and practice to effectively treat fever at home.

## Data Availability

The datasets used and/or analyzed during the current study are available from the corresponding author on reasonable request.
